# Carbapenem-Sparing Strategies for ESBL Producers: When and How

**DOI:** 10.3390/antibiotics9020061

**Published:** 2020-02-05

**Authors:** Ilias Karaiskos, Helen Giamarellou

**Affiliations:** Department of Internal Medicine-Infectious Diseases, Hygeia General Hospital, 15123 Athens, Greece; e.giamarellou@hygeia.gr

**Keywords:** ESBLs, piperacillin–tazobactam, carbapenem-sparing treatment, cefepime, fosfomycin, urinary tract infection

## Abstract

Extended spectrum β-lactamase (ESBL)-producing bacteria are prevalent worldwide and correlated with hospital infections, but they have been evolving as an increasing cause of community acquired infections. The spread of ESBL constitutes a major threat for public health, and infections with ESBL-producing organisms have been associated with poor outcomes. Established therapeutic options for severe infections caused by ESBL-producing organisms are considered the carbapenems. However, under the pressure of carbapenem overuse and the emergence of resistance, carbapenem-sparing strategies have been implemented. The administration of carbapenem-sparing antibiotics for the treatment of ESBL infections has yielded conflicting results. Herein, the current available knowledge regarding carbapenem-sparing strategies for ESBL producers is reviewed, and the optimal conditions for the “when and how” of carbapenem-sparing agents is discussed. An important point of the review focuses on piperacillin–tazobactam as the agent arousing the most debate. The most available data regarding non-carbapenem β-lactams (i.e., ceftolozane–tazobactam, ceftazidime–avibactam, temocillin, cephamycins and cefepime) are also thoroughly presented as well as non β-lactams (i.e., aminoglycosides, quinolones, tigecycline, eravacycline and fosfomycin).

## 1. Introduction

The spread of extended spectrum β-lactamase (ESBL)-producing bacteria has increased the last two decades in the hospital setting as well as in the community, emerging as a serious threat of public health [1]. In particular, infections caused by antimicrobial-resistant *Escherichia coli* proportionally contributed the most to the burden of antimicrobial resistance in Europe, both as number of cases and number of attributable deaths [2]. The population-weighted mean rates of the third-generation cephalosporin resistance in 2018 were 13.1% and 31.7% for *E. coli* and *Klebsiella pneumoniae* isolates, respectively, in the EU and the European Economic Area [2]. ESBLs are enzymes that confer resistance to most beta-lactam antibiotics, including third-generation cephalosporins and monobactams, and they are often seen in combination with other resistance mechanisms, causing multidrug resistance [3]. The majority of ESBLs belong to Ambler class A and include the sulfhydryl reagent variable β-lactamase (SHV), Temoniera β-lactamase (TEM) and cefotaxime-M β-lactamase (CTX-M) types [3]. Infections caused by ESBL-producing Enterobacterales (ESBL-PE) are associated with increased mortality rates, prolonged hospital stays and increased costs [4]. Most clinical factors associated with colonization and infection with ESBL-producing organisms involve healthcare exposure, such as hospitalization, residence in a long-term care facility, hemodialysis use and presence of an intravascular catheter [5,6]. Risk factors for community-acquired infections include recent antibiotic therapy, use of corticosteroids, and the presence of a percutaneous feeding tube as well as international travel [7,8]. Carbapenems have been considered the “gold standard” treatment for the treatment of ESBL-PE and have been associated with improved outcomes, even when in vitro activity to other β-lactams is exhibited [9]. These findings cannot be extrapolated to all patients, as a considerable amount of literature has been published on the use of β-lactams/β-lactamase inhibitor combinations (BLBLI) and specifically piperacillin–tazobactam [10,11,12,13]. In addition, the implementation of carbapenem-sparing strategies has also been applied in ESBL infections in order to combat the overuse of carbapenems and to facilitate antibiotic stewardship programs [14,15,16,17].

The current review is focused on the current state of evidence regarding carbapenem-sparing antibiotic options including non-carbapenem β-lactams as well as non β-lactams options for the treatment of ESBL-PE infections. A narrative review of relevant studies was conducted using the PubMed/MEDLINE, Scopus and Web of Science databases (from 1970 up to January 2020). The keywords used were ESBL, extended spectrum β-lactamases, carbapenem-sparing agents, bacteremia, septic shock, non b-lactams, carbapenems, meropenem, imipenem–cilastatin, ertapenem, β-lactams/β-lactamase inhibitor combinations, piperacillin–tazobactam, ceftolozane–tazobactam, ceftazidime–avibactam, fosfomycin, tigecycline, eravacycline, aminoglycosides and quinolones. Information regarding empiric and definitive therapy of ESBL infections were included and clinical studies comparing the efficacy of carbapenem sparing agents to carbapenems were prioritized. In depth analysis focusing on piperacillin–tazobactam treatment for ESBL infections was covered as this agent has accumulated studies and appears to arouse the greatest debate. Evidence on non β-lactams (i.e., fosfomycin, tigecycline, eravacycline, aminoglycosides and quinolones) is thoroughly discussed, and suggestions on their proper use are indicated.

## 2. Piperacillin–Tazobactam

It is clear that piperacillin–tazobactam (PTZ) among non-carbapenem β-lactams represents the most interesting alternative to carbapenems in the treatment of infections causes by ESBL-PE, as well as for de-escalating carbapenems [18]. Despite the fact that a high percent of ESBL isolates demonstrate in vitro susceptibility to PTZ (current break point according to European Committee on Antimicrobial Susceptibility Testing (EUCAST) ≤8 mg/L, and to Clinical & Laboratory Standards Institute (CLSI) ≤16 mg/L), the significance of PTZ for treating ESBL-PE has remained cloudy. Tazobactam by itself is a potent β-lactamase inhibitor. However, Gram-negative bacteria have the ability to produce concomitantly multiple ESBLs and AmpC β-lactamases, as well as possess other resistance mechanisms such as porin mutations and efflux activation, diminishing the activity of PTZ. On the other hand, tazobactam is influenced by the “inoculum effect” [18].

The clinical studies comparing the efficacy of PTZ versus carbapenems in infections caused by ESBL-PE are depicted in Table 1 [10,11,12,13,19,20,21,22,23,24,25,26,27,28,29,30]. Most comparative studies of PTZ versus carbapenems are retrospective and difficult to be evaluated because of several disagreements [11,12,13,19,20,21,22,24,25,27,28,29,30]. Rodríguez-Baño et al. [10] in 2012 conducted a post hoc analysis of patients with blood stream infection (BSI) due to ESBL-PE derived from 6 published prospective cohorts in Spain. Patients treated either with an active in vitro BLBLI (i.e., amoxicillin-clavulanic acid (AMC) and PTZ) or a carbapenem were compared in 2 cohorts: the empirical therapy cohort (ETC) with 103 patients (AMC 37, PTZ 35, carbapenem 31) and the definitive therapy cohort (DTC) with 174 patients (AMC 36, PTZ 18, carbapenem 120). *E. coli* was isolated in 100%, the source of bacteremia being in 70% urinary or biliary. In 13%, ICU admission at infection onset was necessary, pointing out that most patients were not critically ill. At day 30, mortality rates in the ETC were 9.7% vs. 19.4% and in the DTC 9.3% vs. 16.7% for those given BLBLI and carbapenems respectively (pNS). No association between BLBLI empirical therapy or definitive therapy and increased mortality was observed [10]. Despite the equal clinical validity between the administered antibiotics, the following points seem to compromise the results: (a) only *E. coli* infections were treated, whereas no *K. pneumoniae* isolates with *bla*_SHV_ production, mostly resistant to tazobactam inhibition by definition, were included; (b) “low inoculum” infections (urinary and biliary tract) were mostly treated. It should be pointed out that when the MIC to PTZ was ≤4 mg/L mortality was 4.5%, mounting to 23% in the case of MIC ≥8 mg/L. Based on their results, Rodríguez-Baño et al. [10] suggested that PTZ should be given with safety only in “low inoculum” infections and whenever the MIC is ≤4 mg/L at a dosage schedule of 4.5 g every 6 h.

In the effort to evaluate the efficacy of BLBLI versus carbapenems in patients with a non-urinary source of ESBL-PE bacteremia, Ofer-Friedman et al. [11] performed a multicenter, multinational efficacy analysis from 2008 to 2012 comparing outcomes in patients given a carbapenem (69) versus those treated with PTZ (10). Despite the fact that PTZ was numerically connected with increased 30-day mortality (60% vs. 34%), results were not statistically significant (*p* = 0.1) probably because of the small sample size. However, in terms of 90-day mortality, therapy with PTZ was associated with increased mortality. Therefore, authors suggested carbapenems as superior therapy for ESBL-PE infections.

Subsequently, a retrospective study including 92 BSI caused by cefotaxime non-susceptible *E. coli* and *K. pneumoniae* (producing ESBL or AmpC β-lactamases) has been reported [12]. Definitive monotherapy with a carbapenem (23) (mostly meropenem) was compared to a BLBLI (24) (mostly PTZ with MIC of ≤4 mg/L in 70.7% and 8 mg/L in 29.3%). Comparable outcomes were observed in patients given definitive therapy in terms of all-cause mortality, resolution of systemic Inflammatory Response Syndrome (SIRS), length of stay or BSI relapse, without significant differences in reinfections or colonization with multi-drug resistant (MDR) Gram-negatives or even *Clostridium difficile* infection. Harris et al. [12] concluded that “despite the fact that directed therapy with a BLBLI, when susceptibility is proven, may represent an appropriate carbapenem sparing option, larger studies, adequately powered to detect differences in mortality before such a strategy can be recommended, are required”.

Contrary to the reported studies, Tamma et al. [20] in a retrospective study evaluated PTZ (103 patients) in comparison to a carbapenem (110 patients) in the treatment of 213 patients with ESBL BSI caused by *E. coli*, *K. pneumoniae*, *Klebsiella oxytoca* and *Proteus mirabilis*. A MIC to PTZ ≤16 mg/L was a prerequisite for inclusion in the study. The primary outcome was time to death from the first day of BSI. After the first positive blood culture, there were 17 (17%) deaths in the PTZ group vs. 9 (8%) in the carbapenem group. Covariates independently associated with a higher risk of death by day 14 were higher Pitt bacteremia score and ICU-level care needed on day 1 of BSI. The adjusted risk of death was 1.92 times higher for patients treated empirically with PTZ. In the final conclusion of the study, authors stated that “until more definitive studies are performed, for patients at high risk of invasive ESBL infections, early carbapenem therapy should be considered”. However, a major issue not commented was source control of catheter-related infections as a cause of bacteremia in 43.7% of the included patients [20].

One year later, Gutiérrez-Gutiérrez et al. [13] published an international observational study (called INCREMENT study), investigating the possibility of replacing carbapenems with BLBLI for treating BSI due to ESBL-PE. The main outcomes were clinical response, as cure/improvement at day 14 and 30-day mortality. Two groups of patients were included—(a) 365 in an empirical therapy cohort (PTZ in 123 cases and a carbapenem in 195) and (b) 601 in a targeted therapy cohort (PTZ in 60 and a carbapenem in 509)—therefore comprising, to date, the largest study cohort. The 14-day cure/improvement rates were, in the first cohort, 78.9% for carbapenems and 80% for BLBLIs (*p* = 0.81), with mortality rates of 20% vs. 17.6% (*p* = 0.3), and in the second cohort 90.2% and 85.5% (*p* = 0.22) with mortality rates of 9.8% vs. 13.9% (*p* = 0.28) respectively. The authors concluded that “active in vitro BLBLIs are not inferior to carbapenems for the treatment of BSI due to ESBL-PE in different clinical scenarios”, suggesting that BLBLIs may be useful alternatives to carbapenems if used in appropriate doses [13]. An important issue raising doubts of PTZ efficacy is the diminished activity of tazobactam in the presence of a high burden of bacteria with MICs frequently near the breakpoints, whereas the MICs of carbapenems (except ertapenem) are usually several dilutions below the breakpoints rendering, at least from the pharmacokinetic/pharmacodynamic (PK/PD) aspect, the carbapenems advantageous [31]. On the other hand, the type of pathogens seemed to be influential in mortality rates, since *K. pneumoniae* was independently associated with higher death rate than *E. coli* both in the targeted and the global-therapy cohort [13]. Despite the fact that the INCREMENT data were the best available evidence to support the use of BLBLIs in carbapenem-sparing programs at the time of their publication, prospective randomized control trials to evaluate high-inoculum infections, severe infections as well as Enterobacterales infections with elevated MICs were requested [13].

In a multicenter retrospective cohort study conducted in Singapore, empiric PTZ (94 patients) vs. carbapenems (57 patients) was compared in patients with either ESBL *E. coli* (=101) or ESBL*K. pneumoniae* (=50) BSI [21]. Thirty-day mortality did not differ between the two groups (30.9% vs. 29.8%, *p* = 0.89), whereas PTZ was connected with fewer multidrug-resistant organisms (MDRO) and fungal superinfections compared to carbapenems (7.4% vs. 24.6%, *p* < 0.01). The authors pointed out that for a PTZ MIC of ≤16 mg/L, PTZ is reasonable to be administered at a dose of 3.375 g/8 h with 4 h infusion. Therefore, the mode of administration is important to evaluate when considering the efficacy of PTZ in the comparable arms of therapy [21].

In a prospective randomized open-label comparison trial with a limited number of 64 patients with febrile healthcare-associated complicated urinary tract infections (cUTIs) with ESBL-producing *E. coli*, the efficacy of PTZ was compared to ertapenem [23]. Regarding clinical and microbiological success of PTZ versus ertapenem, the former reached 93.9% vs. 97.0% and 97.0% vs. 97.0% respectively, with a 28-day mortality of 6.1% equal in the two groups. The authors concluded that “PTZ is effective in the treatment of UTIs caused by ESBL-producing *E. coli* whenever the MIC is ≤1 mg/L”.

Similarly, a retrospective observational targeted study of PTZ (68 patients) vs. ertapenem (82 patients) for acute pyelonephritis caused by ESBL-PE (possessing MIC to PTZ of ≤8μg/mL) was performed [24]. No significant difference between PTZ and ertapenem regarding the primary end-points of the study, that is, microbiological eradication failure (4.4% vs. 4.9%), in-hospital mortality (4.4% vs. 13.4%) and change of initial antibiotic regimen (14.7% vs. 22.0%), were observed. In the multivariate analysis, predictors of treatment failure included septic shock and recent administration of immunosuppressive agents; however, the type of the administered antibiotic was not associated with treatment outcome.

The results of two metanalyses referring to carbapenems versus alternative antibiotics for treating ESBL-PE BSI, both published in 2018, are quite interesting [32,33]. In the first metanalysis, 35 publications (until 2016) fulfilled the inclusion criteria [32]. Whenever antibiotics were given empirically, no significant differences related to overall mortality were observed between carbapenems and non-carbapenems. As it concerns definitive therapy, overall mortality was lower for patients given carbapenems compared to cephalosporins and non-BLBLIs, whereas no differences between carbapenems and BLBLIs, as well as quinolones and aminoglycosides, were observed. Despite the absence of differences when BLBLIs were compared to carbapenems, the authors pointed out the lack of robust data derived from randomized controlled trials, as well as the heterogeneity of the study population.

In the second metanalysis, 25 observational studies (until 2017) including 3847 patients were analyzed [33]. Thirty-day mortality of BLBLIs or PTZ was not statistically different from carbapenems either as empirical or definitive therapy. Moreover, the authors suggested that PTZ may be considered as an alternative treatment for ESBL-PE BSI, particularly when the MIC is low (≤4 mg/L) and/or the source of the infection is abdominal or genito-urinary. Sfeir et al. [33] also pointed out the limitations encountered in their review, such as the observational character and the heterogeneity of the studied population, as well as the lack of information on the mode of administration of PTZ (i.e., high dose and continuous 4 h infusion for achieving adequate PK/PDs therapeutic targets [10]). However, the reported limitations should not be an obstacle for suggesting PTZ at high dose and continuous infusion as a non-inferior carbapenem-sparing agent against ESBL-PE. It is of great importance to mention that PTZ is not suitable for deep-seated infections associated with high inoculum (where carbapenems should be preferred), since PTZ possesses a strong inoculum effect leading to ≥8-fold increase in the MIC [34].

The results regarding the efficacy of PTZ in infections caused by ESBL-PE are based mainly on retrospective studies and are controversial (Table 1). The so-called MERINO trial [26,35] was conducted to answer the key question “Can PTZ be used as carbapenem sparing therapy in patients with bloodstream infections caused by ceftriaxone-resistant *E. coli* or *K. pneumoniae* that test susceptible to PTZ and meropenem?”. The MERINO study was an international, multicenter, noninferiority, open-label, parallel group, randomized clinical trial comparing 30-day mortality of PTZ (4.5 g q6h) vs. meropenem (1 h q8h) both infused over 30 min, as definitive therapy in adult patients with ceftriaxone-resistant *E. coli* or *K. pneumoniae* BSI. Randomization was performed within 72 h of blood culture collection, and patients received study drugs for a minimum of 4 d and a maximum of 14 d after randomization with an arbitrary length of treatment arranged by the treating physician. A 5% noninferiority margin was used. Patients were screened for enrollment in 26 hospitals in 9 countries (Australia, New Zealand, Singapore, Italy, Turkey, Lebanon, South Africa, South Arabia and Canada) starting February 2014 until July 2017. Stratification included infecting species, presumed source of infection (UTI or elsewhere) and infection severity (Pitt bacteremia score ≤4 or >4). Primary outcome was all-cause mortality at 30 d post randomization, with secondary outcomes including (a) time to clinical and microbiological resolution of infection; (b) clinical and microbiological success at day 4 post randomization; (c) microbiologic resolution of infection; (d) bloodstream infection relapse; (e) superinfection with meropenem or PTZ-resistant microorganisms or *Clostridium difficile* infections. Finally, out of 1646 screened patients, 378 were randomized (191 in meropenem and 188 in PTZ group). Although balanced with respect to baseline characteristics, more patients in the meropenem group had diabetes, a urinary source of bacteremia and higher APACHE II scores (41.4% vs. 31.4%, 67.0% vs. 54.8% and 21.0% vs. 17.9% respectively), whereas in the PTZ group more patients were immunocompromised (27.1% vs. 20.9% accordingly). A total of 23 patients (12.3%) receiving PTZ vs. 7 (3.7%) in the meropenem group met the primary outcome of 30-day mortality (*p* = 0.90 for noninferiority). In microbiological analysis, a total of 306 isolates were available (266 *E. coli* and 40 *K. pneumoniae*) with median MIC to PTZ of 2 mg/L (IQR 1.5–4 mg/L) and median MIC to meropenem of 0.023 mg/L (IQR 0.016–0.032 mg/L). ESBL genes were confirmed in 85.3% isolates with 10.2% possessing acquired AmpC genes and 2% both. Narrow-spectrum oxacillinases (*bla*_OXA-1_), which may compromise β-lactamase inhibition by tazobactam, were identified in 67.6% of the strains. The authors stated that “PTZ should no longer be considered an alternative to meropenem for definitive treatment of bloodstream infections due to ceftriaxone-resistant *E. coli* and *K. pneumoniae*” [26]. However, certain limitations should be taken into consideration: (a) the inherent delay in blood cultures processing and susceptibility results, indicating that empiric therapy was not throughout under control; (b) the fact that “step down therapy” occurred only in 20.1% of carbapenem-treated patients; (c) crossover of patients from one group to the other was allowed; (d) the lack of information regarding adequate source control; (e) the presence of acquired AmpC in 10.2% of the strains could have an impact on PTZ efficacy, since such enzymes reduce β-lactamase inhibition by tazobactam at least in vitro [9]; (f) due to unblind study design, investigators were aware of the treatment allocation, prompting therefore early cessation of PTZ; (g) the acuity of infection was lower than expected; (h) the high percentage of patients (40.7%) with resolved signs of infection at the randomization day, providing strong evidence against the noninferiority of PTZ [26,35].

Finally, certain questions were left unanswered in the MERINO trial [26]. Questions that need to be answered, however, are whether PTZ given in extended or constant infusion is efficacious, as well as the effectiveness of PTZ in cases of empirical therapy of bacteremia or in the treatment of non-bacteremic ESBL-PE infections. Probably, a European plus a USA blinded trial (similar to MERINO), taking into account the reported limitations analyzed, could give answers to the existing questions. In the meantime, it seems preferable, whenever a carbapenem-sparing decision is pending, to seriously consider the Tamma and Rodríguez-Baño et al. [18] positions.

## 3. Ceftolozane–Tazobactam

Ceftolozane–tazobactam is a novel combination of a cephalosporin with a β-lactamase inhibitor that exhibits excellent in vitro activity against a broad spectrum of Enterobacterales and *Pseudomonas aeruginosa*, including ESBL strains, and has been recently approved for the treatment of complicated intra-abdominal infections (cIAI) and cUTI [36]. The in vitro activity of ceftolozane–tazobactam against ESBL-PE from U.S. hospitals revealed an overall susceptibility of 83.9% (CLSI/EUCAST breakpoints) to ceftolozane–tazobactam. Ceftolozane–tazobactam inhibited 95.5% of the *E. coli* isolates, but only 83% of *K. pneumoniae* producing ESBL. Regarding ESBL-encoding genes, ceftolozane–tazobactam inhibited 92.9% of the isolates harboring bla_CTX-M_ and exhibited limited activity against isolates carrying bla_SHV_ (61.1% susceptible) [37]. In phase III randomized clinical trials, ceftolozane–tazobactam (in combination with metronidazole) demonstrated similar efficacy to meropenem for the treatment of cIAIs [38] and superior efficacy to levofloxacin for the treatment of cUTIs, including pyelonephritis [39]. Also, in a phase 3 trial with nosocomial infections, ceftolozane–tazobactam was compared to meropenem and in patients with ESBL-PE, and clinical cure rates were 57.1% (48/84) and 61.6% (45/73) respectively [40]. In a pooled analysis of phase III clinical trials, a total of 159 ESBL-PE isolates from the microbiologically evaluable population, mostly *E. coli* (68.6%), were identified. Overall, 72.3% of ESBL isolates were susceptible to ceftolozane–tazobactam versus 98.3% to meropenem, whereas only 24.1% were susceptible to levofloxacin at EUCAST breakpoints. Clinical cure rates against ESBL isolates in cUTIs treated with ceftolozane–tazobactam and levofloxacin were 95.8% and 82.6% respectively, whereas in cIAIs, clinical cure rates depicted were 98.1% for the ceftolozane–tazobactam group and 88.5% for the meropenem group [41]. It is of great significance to point out that, in a cost effectiveness analysis comparing carbapenem-sparing agents versus meropenem, patients with cUTIs due to ESBL receiving ceftolozane–tazobactam were found cost-effective compared to meropenem [42]. Although the limited data available for ESBL pathogens are extrapolated from clinical trials to preclude robust analysis, ceftolozane–tazobactam seems as an attractive option for a carbapenem-sparing strategy depending on local antibiotic stewardship decisions. However, more evidence is needed to confirm the exact place of ceftolozane–tazobactam for ESBL infections.

## 4. Ceftazidime–Avibactam

Avibactam, a novel non-b-lactam, b-lactamase inhibitor, restores the activity of ceftazidime against the majority of β-lactamases (ESBLs and carbapenemases, including *Klebsiella pneumoniae* carbapenemase (KPCs) and OXA-48), resulting in activity of ceftazidime–avibactam combination against a wide range of MDR Gram-negative bacteria [43,44]. In vitro activity of ceftazidime–avibactam against Enterobacterales from 18 European countries as part of the International Network for Optimal Resistance Monitoring (INFORM) global surveillance program from 2012 to 2015 revealed ceftazidime–avibactam was the most active agent, compared with all other tested comparator agents, against non-susceptible ceftazidime isolates (97.7% susceptible) [45]. A post hoc analysis of phase 2 trials summarizing the results of ESBL-PE isolates recovered at baseline revealed a favorable outcome in the ceftazidime–avibactam and meropenem arms in 85.7% and 80.0% of patients, respectively [46]. Similarly, clinical cure rates from patients enrolled in phase 3 clinical trial regarding cUTIs revealed efficacies of 90.3% and 89.1% for the ceftazidime–avibactam and doripenem groups accordingly [47], highlighting a potential role for ESBL infections. However, it should be taken into consideration that ceftazidime–avibactam is one of the limited options for carbapenemase-producing Enterobacterales [44]. Therefore, it should be preserved for the treatment of KPC and OXA-48 producers and should not be recommended as a carbapenem-sparing strategy.

## 5. Cephamycins

Cephamycins include cefoxitin, cefotetan, cefmetazole, moxalactam and flomoxef. They belong to a subclass of second-generation cephalosporins that confers resistance to degradation by ESBL enzymes. However, cephamycins are not active against AmpC cephalosporinases and porin mutations [48]. Eight observational studies comparing the efficacy of cephamycins and carbapenems in infections (mainly UTIs and bacteremia) due to ESBL-PE have been published to date and are illustrated in Table 2 [49,50,51,52,53,54,55,56]. In the majority of the studies, no difference regarding mortality was observed [49,51,52,53,55,56] with the exception of two studies [50,54]. The major drawbacks of the studies were the small population size, inadequate control for confounders and low statistical power, whereas carbapenems were administrated in more severe infections and mostly in bacteremic patients [49,50,51,52,53,54,55,56]. Lee et al. [54] found similar mortality rates with flomoxef and carbapenems when the MIC of flomoxef was ≤1 mg/L, but when MIC was 4–8 mg/L, cefoxitin was found inferior to carbapenem. Also, cefoxitin has been studied in UTIs in males. Twenty-three patients were included in the cefoxitin group and 27 in the carbapenem group. No significant difference was illustrated for clinical and microbiological success between the two groups in univariate and multivariable models. An interesting point was that cefoxitin administrated at high dose and continuous infusion was associated with clinical success [56].

The available data suggest that cephamycins may be an alternative to carbapenems for some non-severe infections, particularly UTIs, and should be administrated at high dose and continuous infusion.

## 6. Cefepime

Cefepime, a “fourth-generation” cephalosporin, possesses in vitro activity against most Gram-negative pathogens, including Enterobacterales, due in part to its relatively low susceptibility to degradation by chromosomal and plasmid-mediated extended-spectrum AmpC lactamases and ESBLs compared to that of other cephalosporins [57]. CLSI breakpoints for cefepime MIC against the Enterobacterales family are 2 mg/L (susceptible), 4–8 mg/L (susceptible dose dependent; SDD) and 16 mg/L (resistant) [58]. In contrast, EUCAST breakpoints for cefepime MIC are 1 mg/L (susceptible) and 4 mg/L (resistant) [59]. However, MICs of cefepime for Gram-negative organisms that produce ESBLs are often increased compared to non-producers [57]. A retrospective study by Bhat et al. [60] provided an important opportunity to advance our knowledge regarding the rate of mortality in correlation to cefepime MIC. The rates of mortality were 23.3% and 27.8% for infections with a cefepime MIC of <1 mg/L and 2 mg/L respectively, mounting to 53.3% and 56.3% with a MIC of 8 and 16 g/mL accordingly, highlighting the use of cefepime for non-severe infections only with MIC ≤ 2 mg/L [60].

On the other hand, concerns of diminished efficacy of cefepime for the treatment of ESBL infections with high bacterial inoculum (i.e., pneumonia, osteoarthritis and endocarditis, so called “inoculum effect” in which there is a marked increase in MIC with increased inoculum), has been illustrated in animal studies [34,61,62]. In order to highlight this phenomenon, inkjet printing technology testing the inoculum effect on cefepime was performed in a recent study with ESBL *Escherichia coli* and *Klebsiella* spp. In 13 cefepime-resistant and SDD strains, as well as 11 cefepime-susceptible isolates, a 2-fold increase in inoculum resulted in a 1.6 log_2_-fold increase in MIC. In contrast, in cefepime-susceptible, non-ESBL producing clinical strains, only a minor inoculum effect at very high inocula (>10^7^ cfu/mL^−1^) was depicted [63].

Although some observational data have suggested that high-dose cefepime (e.g., 2 g every eight hours) can be effective for the treatment of ESBL-producing infections as a carbapenem-sparing agent [64,65], superiority of carbapenem treatment has been highlighted regarding therapy of ESBL infections [66,67,68,69]. The clinical studies comparing the efficacy of cefepime versus carbapenems in infections caused by ESBL-PE are illustrated in Table 3 [23,27,64,65,66,67,68,69,70,71]. Chopra et al. [66] outlined in a multivariate analysis that empirical therapy with cefepime for BSI due to an ESBL-producing pathogen was associated with a trend toward an increased mortality risk, and empirical carbapenem therapy was associated with a trend toward decreased mortality risk. Likewise, in a retrospective study of 178 patients with monomicrobial bacteremia caused by ESBL producers, cefepime was administrated in 17 cases compared with 161 patients treated with a carbapenem. Multivariable analysis manifested that cefepime therapy was independently associated with a poor outcome. Moreover, increased risk of clinical and microbiological failure as well as sepsis-related mortality was illustrated with cefepime therapy. However, an interesting finding was the favorable outcome in patients treated with cefepime with bacteremia caused by ESBL-producing organisms only when cefepime MIC was ≤1 mg/L [67]. Similarly, in a randomized trial of patients with UTIs caused by ESBL-PE, treatment failure with cefepime was higher compared with ertapenem or piperacillin–tazobactam [23]. Wang et al. [68] conducted a propensity score-match study of 17 patients receiving empiric cefepime therapy versus 51 patients receiving carbapenem therapy for ESBL bacteremia. Patients receiving carbapenem were 2.87 times more likely to survive than patients receiving cefepime. The dilemma of carbapenem-sparing strategies in bacteremic patients caused by ESBL-producing *E. coli* with hematologic malignancies has been evaluated in a retrospective study comparing carbapenems (42 pts) vs. cefepime (40 pts). In multivariate analysis, empiric treatment with cefepime was not associated with increased 14-day or 30-day mortality. However, time to defervescence (*p* < 0.01) and persistent bacteremia (*p* = 0.07) were associated with cefepime treatment, hindering the use of cefepime in this specific population [27].

In conclusion, the existing literature suggests that cefepime may be suboptimal for invasive ESBL infections. The use of cefepime for serious infection is not safely recommended and has been related to high mortality risk. However, cefepime could be suggested for low-risk infections caused by ESBL (i.e., UTIs with the supposition of a MIC to cefepime ≤2 mg/L administrated at a high dose 2 g every 8 h as a prolonged infusion).

## 7. Temocillin

Temocillin is a b-a-methoxy-derivative of ticarcillin that is only available in UK, Belgium, France and Luxembourg [72]. The chemical modification of ticarcillin to temocillin increased its stability to β-lactamases. Temocillin possesses very attractive characteristics: narrow spectrum mainly restricted to Enterobacterales, high resistance to hydrolysis by numerous β-lactamases including ESBL and hyperproduced AmpC and minimal risk of *Clostridium difficile* infection [72]. Against a collection of 157 ESBL-producing *E. coli* and 95 ESBL-producing *K. pneumoniae* strains harvested in 2015 from three French centers, in vitro susceptibility to temocillin was observed in 71.3% of the ESBL-producing *E. coli* and in 77.9% of the ESBL-producing *K. pneumoniae* tested, according to the Antibiogram Committee of the French Society for Microbiology guidelines. These rates increased to 98.7% and 98.9% according to the urinary tract infection breakpoint (i.e., 32 mg/L) of the British Society for Antimicrobial Chemotherapy [73]. Similarly, susceptibility rate was 94.6% according to the 8 mg/L clinical breakpoint against ESBL-producing isolates from community-acquired UTI [74]. The largest experience on the efficacy of temocillin refers to a retrospective study in 92 patients in whom the drug was given for UTIs, bacteremia and hospital-acquired pneumonia (HAP) caused by Enterobacterales-producing ESBL and/or derepressed AmpC b-lactamases. Clinical and bacteriological efficacies of 86% and 84% were reported. However, the significance of an optimal therapeutic regimen of 2 g every 12 h was pointed out since clinical efficacy and microbiological eradication dropped significantly when suboptimal doses were administrated [75]. On the other hand, a dosage of temocillin of 6 g daily given by continuous infusion in critically ill patients from a PK/PDs aspect managed to cover infections caused by Enterobacterales with an MIC up to 16 mg/L [76]. Taking into consideration cost analysis, temocillin was found cost effective in patients with cUTIs due to ESBL compared to carbapenems, even when a three-year Markov model was applied [42]. Moreover, the impact on intestinal colonization resistance regarding temocillin was marginal, whereas carbapenems, and specifically ertapenem, were found to be a stronger driver of intestinal dysbiosis and colonization resistance alteration [77]. Concluding, temocillin, with its rather unique characteristics may act as an alternative to carbapenems for the treatment of ESBL Enterobacterales infections such as systemic UTIs. However, large-scale randomized trials are needed to shed light on the true efficacy of temocillin for the treatment of serious infections caused by ESBL-PE.

## 8. Quinolones

Plasmid-mediated quinolones resistance (PMQR) determinants frequently appear associated with extended-spectrum ESBL genes [78]. A study from Canada including outpatients revealed quinolone resistance of 18.2% in urinary ESBL-positive *E. coli* [79], whereas in a study from Spain the prevalence of PMQR genes were found in 28.6% [80], limiting the activity of quinolones for the treatment of ESBL infections. A systemic review and meta-analysis illustrated that quinolones were not associated with increased mortality regarding empirical therapy and in relation to definitive treatment [32]. Limited data are available regarding the administration of fluoroquinolones as carbapenem-sparing agents. In a study from Taiwan, 299 bacteremia caused by ESBL Enterobacterales indicated a 30-day mortality rate significantly lower in the quinolone group (8.3%) compared to the carbapenem group (23%) [81]. In contrast, imipenem was found superior to quinolone treatment regarding BSI infections caused by ESBL-producing *K. pneumoniae* [82], whereas in 186 BSI caused by ESBL-PE, a significant predictor of mortality was inadequate initial antimicrobial therapy mainly associated with quinolone treatment [83]. Also, an interesting finding was that inadequately treated patients had a threefold increase in mortality compared to the adequately treated group [83]. Quinolones, as an oral option as well as trimethoprim/sulfamethoxazole (as non-intravenous carbapenem-sparing antibiotic) have been implemented as an alternative to carbapenems for bacteremia caused by ESBL. In a 12-year retrospective study from Spain, the use of alternative non-intravenous therapeutic options was associated with shorter hospital stay with no difference in mortality [84]. Therefore, quinolones could be useful as oral switch whenever susceptible; however, the recent FDA warning on quinolones toxicity should be taken under serious consideration [85].

## 9. Aminoglycosides

Aminoglycosides have been shown to be active against Gram-negative bacteria, including ESBL pathogens [86]. In vitro susceptibility rates may vary significantly, depending on the dissemination of aminoglycoside modifying enzymes, which are frequently co-transferred along with other resistance genes on mobile genetic elements. The presence of genes encoding aminoglycoside-modifying enzymes with genes located on integrons or transposons also coding for ESBLs have been reported [87]. Amikacin has been shown to be the most active aminoglycoside against ESBL-PE [88]. The advantageous pharmacokinetic parameter of the aminoglycosides consisting of high urine concentrations, therefore, have been utilized in urinary tract infections as monotherapy [43,89]. In the INCREMENT ESBL cohort analysis, empiric treatments with carbapenem-sparing agents (mainly aminoglycosides and fluoroquinolones) versus carbapenem therapy in 86 and 245 patients were included, respectively [90]. No significant differences regarding 30-day mortality between patients treated empirically with carbapenems or aminoglycosides were observed [90]. Even when a stratified analysis was also performed using Kaplan–Meier curves on the low-risk (<11 points) and high-risk (≥11) strata of the INCREMENT ESBL score [91], no significant differences on mortality were depicted between the two groups [90]. Similarly, in a retrospective study from Israel, aminoglycoside monotherapy was compared to carbapenem or PTZ for the treatment of ESBL-PE in cUTI associated or not with secondary bacteremia. Aminoglycoside treatment was found non-inferior compared with other antibiotics regarding 30-day mortality; however, non-inferiority for bacteriuria recurrence was observed. An important issue to be mentioned was that patients treated with an aminoglycoside had less severe disease, lower burden of chronic illness and lower incidence of septic shock [92]. In a recent meta-analysis, aminoglycoside treatment was not associated with lower mortality rates regarding empiric and definite treatment compared to carbapenems for the treatment of ESBL-PE bacteremia [32]. Recently, plazomicin, a next-generation aminoglycoside synthetically derived from sisomicin designed to evade all clinically relevant aminoglycoside-modifying enzymes, was launched in market [93]. Plazomicin has been approved by FDA for the treatment of cUTI including acute pyelonephritis [94]. In a phase 3 study, plazomicin was compared to meropenem for the treatment of cUTI. ESBL-PE were present in 57 of 197 patients treated with meropenem and 50 out of 191 treated with plazomicin. Clinical cures at test of cures were 69% and 72% respectively [94]. In conclusion, aminoglycosides seem to be an attractive alternative for the treatment of UTIs caused by ESBL-PE (depending on local epidemiology susceptibility patterns).

## 10. Tigecycline–Eravacycline–Omadacycline

Tigecycline, a glycylcycline, is a bacteriostatic derivative of minocycline [72]. It was approved by the FDA and the European Medicines Agency (EMA) in 2005 and 2006, respectively, for the treatment of cIAIs and complicated skin and skin structure infections, and the FDA in 2009 added community-acquired pneumonia to the list. However, nowadays tigecycline is frequently administered off-label for treating MDR infections [43,72]. Tigecycline antimicrobial spectrum includes ESBL-PE, MDR and extensively-drug resistant (XDR) *Acinetobacter baumannii* and *K. pneumoniae* [43,72]. From the Tigecycline Evaluation and Surveillance Trial, antimicrobial susceptibility of ESBL-producing *E. coli* to tigecycline from clinically relevant isolates revealed very low resistance rates below 2% [95]. Treatment outcomes have been hampered by the low serum concentrations of the drug in the approved dosing regimen, the low penetration in the epithelial lining fluid as well as the low concentrations achieved in the urinary system [96]. Tigecycline has been administrated for IAI and soft tissue infections caused by ESBL-producing isolates in non-comparative studies with high clinical cure rates [97,98]. Importantly, both the FDA and the EMA issued warnings because the drug was associated with an increased risk of mortality and clinical failure in meta-analyses of randomized trials [99,100]. Moreover, a basic drawback is a significant number of adverse events (i.e., nausea, vomiting, pancreatitis, hepatotoxicity [72]) as well as more severe ones, consisting of decreased fibrinogen levels and alterations in coagulation parameters [101]. Eravacycline and omadacycline are novel tetracycline derivatives with significant activity against ESBL Gram-negative bacilli [102,103]. Eravacycline has recently been approved by FDA and EMA for adult patients with cIAI [104,105]. In phase 3 with cIAI, participants were randomized to either eravacycline 1 mg/kg q12 h or meropenem 1 g q8 h, both given intravenously. In patients with ESBL-PE, clinical cure rates were 88% (15/17) and 81% (13/16) in the eravacycline and meropenem groups, respectively [106]. In conclusion, tigecycline is not indicated as a suitable alternative for the treatment of ESBL infections because of several limitations as reported above. However, tigecycline therapy could be appropriate for soft tissue infections and IAI caused by ESBL-PE. Eravacycline is a promising agent for carbapenem-sparing strategies (is available intravenously and also in an oral formula) for the treatment of cIAI caused by ESBL-PE with fewer drawbacks compared to tigecycline. On the other hand, omadacycline could be suitable for soft tissue infections.

## 11. Fosfomycin

Fosfomycin inhibits phosphoenolpyruvate transferase, the first enzyme involved in the synthesis of peptidoglycan. It possesses advantageous pharmacokinetics mainly in the urinary tract system as well as in cerebrospinal fluid, lungs, bone and soft tissue [72]. Fosfomycin is active against a broad spectrum of Gram-positive and Gram-negative bacteria, including ESBL isolates and possesses a low potential for cross resistance with other classes of antibiotics. The relevant available formulation for intravenous administration is fosfomycin disodium. The current recommended intravenous dosage ranges from 16–24 g daily [43,72]. In a review of 11 studies, among 5057 isolates of Enterobacterales, including *E. coli* (2205 strains) and *K. pneumoniae* (764 strains), 88% of which produced ESBL, 91.3% were found susceptible to fosfomycin [107]. In a real-world perspective and review of the literature of fosfomycin for the treatment of MDR-causing UTIs, patients infected by ESBL-PE demonstrated rates of clinical success of 56% [108]. The results of a multicenter, randomized, double-blind phase 2/3 trial were announced, showing non-inferiority of intravenous fosfomycin to PTZ, in patients with cUTIs or acute pyelonephritis [109]. The most notable finding of most studies with MDR UTIs treated with fosfomycin monotherapy was the high rates of clinical and microbiologic cure encountered [108,109]. However, no current evidence regarding efficacy of intravenous fosfomycin versus carbapenem is existent. The FOREST trial, a phase 3 clinical trial, has been completed (but not yet analyzed) to prove the non-inferiority of fosfomycin versus meropenem in the targeted treatment of bacteremic UTI due to ESBL-PE, in order to render the elucidation of the true efficacy of intravenous fosfomycin in the era of MDR pathogens [110].

Fosfomycin tromethamine, a soluble salt of fosfomycin, represents the oral formula. Fosfomycin’s in vitro activity against common uropathogens, including ESBL isolates, its favorable safety profile, including pregnancy patients, and clinical trial data demonstrating efficacy in cystitis have resulted in recommending fosfomycin as a first line agent for the treatment of acute uncomplicated cystitis [111]. Recently, oral fosfomycin in a unique dosage scheme of 3 g daily for 1 w followed by 3 g every 48 h for a total of 6‒12 w has been promising for the treatment of chronic bacterial prostatitis, including ESBL isolates [112]. Fosfomycin appears as an attractive carbapenem-sparing option mainly for the treatment of cUTI. However, the results of randomized trials are pending to ensure the true efficacy on fosfomycin for the therapy of ESBL UTIs.

## 12. Conclusions

The established therapeutic option for severe infections caused by ESBL-PE is a carbapenem; however, ertapenem could be an acceptable option in the absence of severe sepsis or resistance. On the other hand, PTZ appears to be a possible option for low- to moderate-severity infection, originating from urinary or biliary sources and when PTZ MIC ≤ 4 mg/L. Ceftolozane–tazobactam appears promising, but further clinical data are needed to establish its efficacy relative to carbapenems. Fosfomycin, aminoglycosides and temocillin are relatively effective for cUTI and could replace carbapenems for the treatment of ESBL-PE. There is little clinical evidence for cephamycin use, which has been associated with development of resistance. Resistance to fluoroquinolones is nowadays common in ESBL-PE. Cefepime may be effective against ESBL-producing organisms that test susceptible (with an MIC ≤ 2mg/L) if administered in high doses (i.e., 2 g every 8 h); however, it has been associated with increased mortality risk.

## Figures and Tables

**Table 1:** Clinical studies comparing the efficacy of piperacillin–tazobactam versus carbapenems in infections caused by ESBL-producing Enterobacterales [10,11,12,13,19,20,21,22,23,24,25,26,27,28,29,30].

Study	Country of Study (Period of Study)	Study Design	PTZ (*n*, Number of Participants)	Carbapenems (*n,* Number of Participants)	Organism(s)	Site of Infection	Severity of Illness at Infection Onset	Outcome (PTZ vs Carbapenems)	Comments
Rodríguez-Baño et al. ^a^ [10]	Spain (2001–2006)	Post hoc analysis of 6 prospective cohorts	Empiric: *n* = 35 Definitive: *n* = 18	Empiric: *n* = 31 Definitive: *n* = 120	*Escherichia coli* (100%)	BSI (100%) -urinary or biliary (70%)	ICU: 13% Severe sepsis or shock: 23%	**30-day mortality** (empiric): 10% vs 19% (ns) **30-day mortality** (definitive): 9% vs 17% (ns)	No association between either empirical or definitive therapy with PTZ and increased mortality
Kang et al. [19]	Korea (2008–2010)	Retrospective	*n* = 36	*n* = 78	*E. coli* (68%) *Klebsiella pneumoniae* (32%)	BSI (100%)	NR	**30-day mortality**: 22% vs 27% (ns)	No difference between PTZ and carbapenem treatment
Tamma et al. [20]	USA (2007–2014)	Retrospective	*n* = 103	*n* = 110	*K. pneumoniae* (68%) *E. coli* (31%) *Proteus mirabilis* (1%)	BSI (100%) -CRBSI (46%) -UTI (21%) -cIAI (17%) -Biliary (9%) -pneumonia (9%)	ICU:34% Neutropenia: 15%	**14-day mortality**: 17% vs 8% (*p* < 0.05) **30-day mortality**: 26% vs 11% (*p* < 0.01)	PTZ inferior to carbapenems for the treatment of ESBL bacteremia. Risk of death 1.92 times higher for patients on empiric PTZ therapy
Ofer-Friedman et al. [11]	Multicenter (USA, Israel) (2008–2012)	Retrospective	*n* = 10	*n* = 69	*E. coli* (53%) *K. pneumoniae* (28%) *P. mirabilis* (19%)	BSI (100%) -pneumonia (34%) -SSTI (28%) -Biliary (17%) -cIAI (9%)	Rapid fatal condition per McCabe score: 39%	**30-day mortality**: 60% vs 34% (*p* = 0.10) **90-day mortality**: 80% vs 48% (*p* = 0.05)	Therapy with PTZ was associated with increased 90-day mortality (adjusted OR, 7.9. *p* = 0.03)
Harris et al. [12]	Singapore (2012–2013)	Retrospective	*n* = 24	*n* = 23	*E. coli* (86%) *K. pneumoniae* (14%)	BSI (100%) -UTI (47%) -Biliary (9%)	ICU: 15%	**30-day mortality**: 8% vs 17% (ns)	No difference between PTZ and carbapenem treatment
Gutiérrez-Gutiérrez et al. ^a^ [13]	INCREMENT international project (2004–2013)	Retrospective	Empiric: *n* = 123 Definitive: *n* = 60	Empiric: *n* = 195 Definitive: *n* = 509	*E. coli* (73%) *K. pneumoniae* (19%)	BSI (100%) -UTI (45%) -Biliary (12%)	ICU: 11% Severe sepsis or shock: 32%	**30-day mortality** (empiric): 18% vs 20% (ns) **30-day mortality** (definitive): 10% vs 14% (ns)	No association between either empirical or definitive therapy with PTZ and increased mortality
Ng et al. [21]	Singapore (2011–2013)	Retrospective	*n* = 94	*n* = 57	*E. coli* (67%) *K. pneumoniae* (33%)	BSI (100%) -UTI (59%) -Biliary (9%) -Pneumonia (9%) -cIAI (5%) -CRBSI (4%)	ICU: 9%	**30-day mortality**: 31% vs 30% (ns)	No difference between PTZ and carbapenem treatment
Gudiol et al.^a^ [22]	Multicenter (2006–2015)	Retrospective	Empiric: *n* = 44 Definitive: *n* = 12	Empiric: *n* = 126 Definitive: *n* = 234	*E. coli* (74%) *K. pneumoniae* (23%) *K. oxytoca* (1.5%) *Enterobacter cloacae* (1.5%)	BSI (100%) -Primary (53%) -CRBSI (18%) -cIAI (15%) -UTI (7%)	ICU: 18% Septic shock: 22% Hematological neutropenic patients: 100%	**30-day mortality** (empiric): 21% vs 13% (ns) **30-day mortality** (definitive): 6% vs 16% (ns)	PTZ appeared to have similar efficacy to carbapenems in hematological neutropenic patients
Seo et al. [23]	Korea (2013–2015)	Randomized trial	*n* = 33	*n* = 33	*E. coli* (100%)	UTI (100%) BSI (11%)	Septic shock: 30%	**28-day mortality**: 6.1% vs 6.1% (ns)	PTZ appeared to have similar efficacy to ertapenem in UTIs
Yoon et al. [24]	Korea (2011–2013)	Retrospective	*n* = 68	*n* = 82	*E. coli* (100%)	UTI (100%) BSI (15%)	ICU: 25% Septic shock: 16%	**In-hospital mortality**: 4.4% vs 13% (ns)	PTZ appeared to have similar efficacy to ertapenem in UTIs
Ko et al. ^a^ [25]	Korea (2010–2014)	Retrospective	*n* = 41	*n* = 183	*E. coli* (66%) *K. pneumoniae* (34%)	BSI (100%) -Primary (24%) -CRBSI (3%) -UTI (37%) -cIAI (28%)	ICU: 33%	**30-day mortality**: 6.3% vs 11.4% (ns)	No difference between PTZ and carbapenem treatment
Harris et al. [26]	International, multicenter (2014–2017)	Randomized trial	*n* = 188	*n* = 191	*E. coli* (87%) *K. pneumoniae* (13%)	BSI (100%) - UTI (61%) -cIAI (16%) -CRBSI (2%) -Pneumonia (3%) -Mucositis (5%) -SSTI (1%)	ICU: 7% Neutropenia: 7%	**30-day mortality**: 12.3% vs 3.7% (*p* = 0.90)	Definitive treatment with PTZ compared with meropenem did not result in a non-inferior 30-day mortality
Benanti et al. [27]	USA (2008–2015)	Retrospective	*n* = 21	*n* = 42	*E. coli* (100%)	BSI (100%) - cIAI (40%) -UTI (10%) -CRBSI (11%) -Pneumonia (11%) -SSTI (10%)	ICU: 30% Neutropenia: 89%	**14-day mortality**: 0% vs 19% (*p* = 0.04)	Empiric treatment with PTZ not associated with increased mortality in patients with hematologic malignancy
John et al. [28]	USA (2014–2017)	Retrospective	*n* = 66	*n* = 51	*E. coli* (86%) *K. pneumoniae* (14%)	BSI (100%) -UTI (73%) -cIAI (19%) -Pneumonia (1%)	ICU: 38% Septic shock:17%	**In-hospital mortality**: 3% vs 7.8% (ns)	PTZ appeared to have similar efficacy to carbapenems
Nasir et al. ^a^ [29]	Pakistan (2015–2017)	Retrospective	*n* = 89	*n* = 174	*E. coli* (100%)	BSI (100%) -UTI (66%) -cIAI (23%) -CRBSI (3%)	ICU: 38% Septic shock:17%	**In-hospital mortality**: 13% vs 21% (ns)	PTZ appeared to have similar efficacy to carbapenems
Sharara et al. [30]	USA (2014–2016)	Retrospective	*n* = 45	*n* = 141	*E. coli* (56%) *K. pneumoniae* (30%) *P. mirabilis* (10%) *K. oxytoca* (4%)	UTI (100%)	ICU: 26%	**30-day mortality**: 4% vs 7% (ns)	PTZ appeared to have similar efficacy to carbapenems. Patients treated with carbapenem had higher incident of carbapenem-resistant organism isolated in 60 d (*p* = 0.09)

BSI, blood stream infection; CRBSI, catheter-related blood stream infection; cIAI, complicated intra-abdominal infection; ESBL, extended spectrum β-lactamases; ICU, intensive care unit; NR, not reported; ns, not significant; OR, odds ratio; PTZ, piperacillin–tazobactam; SSTI, skin and soft tissue infections; UTI, urinary tract infection; vs, versus. ^a^ Studies including β-lactam/β-lactamase inhibitors.

**Table 2:** Clinical studies comparing the efficacy of cephamycins versus carbapenems in infections caused by ESBL-producing Enterobacterales [49,50,51,52,53,54,55,56].

Study	Country of Study (Period of Study)	Study Design	Cephamycin (*n*, Number of Participants)	Carbapenems (*n*, Number of Participants)	Organism(s)	Site of Infection	Severity of Illness at Infection Onset	Outcome (Cephamycins vs Carbapenems)	Comments
Lee et al. [49]	Taiwan (2004–2005)	Retrospective	*n* = 7 (flomoxef)	*n* = 20	*K. pneumoniae* (100%)	BSI (100%)	ICU: 52%	**14-day mortality**: 29% vs 25% (ns)	No difference between cephamycin and carbapenem treatment. Patients in the carbapenem group were more severely ill
Yang et al. [50]	Taiwan (2001–2007)	Retrospective	*n* = 29 (flomoxef)	*n* = 28	*K. pneumoniae* (100%)	BSI (100%)	ICU: 51%	**14-day mortality**: 55% vs 39% (p < 0.05)	Hemodialysis access-related bacteremia included in the study. Cephamycin use was independently associated with increased mortality (OR, 3.52; 95% CI, 1.19–58.17)
Doi et al. [51]	Japan (2008–2010)	Retrospective	*n* = 10 (cefmetazole)	*n* = 12	*E. coli* (95%) *K. pneumoniae* (5%)	UTI (100%)	NR	**Clinical cure** (4-weeks): 90% vs 100% (ns)	No difference in clinical or bacteriological cure rate at 4 w
Pilmis et al. [52]	France (2011)	Retrospective	*n* = 8 (cefoxitin)	*n* = 31	*E. coli* (32%) *K. pneumoniae* (32%) *E. cloacae* (36%)	UTI (75%) BSI (25%)	NR	**Clinical and microbiological relapse** (30-days): 13% vs 23% (ns)	No difference between cephamycin and carbapenem treatment
Matsumura et al. [53]	Japan (2005–2014)	Retrospective	Empiric, *n* = 8 Definitive, *n* = 59 (cefmetazole or flomoxef)	Empiric, *n* = 45 Definitive, *n* = 54	*E. coli* (100%)	BSI (100%)	Septic shock: 41%	**30-day mortality** (empiric arm): 8% vs 9% (ns) **30-day mortality** (definitive arm): 5% vs 9% (ns)	No difference between cephamycin and carbapenem treatment
Lee et al. [54]	Taiwan (2007–2012)	Retrospective	*n* = 123 (flomoxef)	*n* = 257	*K. pneumoniae* (60%) *E. coli* (40%)	BSI (100%)	Pitt bacteremia score ≥4: 66%	**30-day mortality**: 28.8% vs 12.8%	Definitive flomoxef therapy appears to be inferior to carbapenems, particularly for isolates with a MIC flomoxef of 2–8 mg/L
Fukuchi et al. [55]	Japan (2008–2013)	Retrospective	*n* = 26 (cefmetazole)	*n* = 43	*E. coli* (94%) *K. pneumoniae* (3%) *K. oxytoca* (3%)	BSI (100%)	ICU: 32%	**30-day mortality**: 4% vs 16% (ns)	No difference between cephamycin and carbapenem treatment. The group that received carbapenem therapy had increased severity
Senard et al. [56]	France (2013–2015)	Retrospective	*n* = 23 (cefoxitin)	*n* = 27	*E. coli* (100%)	UTI (100%)	Septic shock: 4%	**Clinical success**: 73.9% vs 81.5% (ns) **Clinical success**: 57.9% vs 50% (ns)	No difference between cephamycin and carbapenem treatment. In the cephamycin group, continuous infusion was associated with clinical success

BSI, blood stream infection; CI, confidence interval; ICU, intensive care unit; NR, not reported; ns, not significant; OR, odds ratio; UTI, urinary tract infection; vs, versus.

**Table 3:** Clinical studies comparing the efficacy of cefepime versus carbapenems in infections caused by ESBL-producing Enterobacterales [23,27,64,65,66,67,68,69,70,71].

Study	Country of Study (Period of Study)	Study design	Cefepime (*n*, Number of Participants) (Dosage)	Carbapenems (*n*, Number of Participants) (Dosage)	Organism(s)	Site of Infection	Severity of Illness at Infection Onset	Outcome (Cefepime vs Carbapenems)	Comments
Zanetti et al. [64]	Six European countries (1997–1999)	Randomized trial	*n* = 13 (2 gr q8h)	*n* = 10 (IMP 500 mg q6h)	*K. pneumoniae* (96%) *E. aerogenes* (4%)	Pneumonia (100%)	ICU (100%)	**Clinical response**: 69% vs 100% (*p* < 0.05)	Comparison for ESBL producers not included
Goethaert et al. [65]	Belgium (1994–2000)	Retrospective	*n* = 21 (2 gr q8h)	*n* = 23 (IMP 500 mg q6h, MEM 1 gr q8h)	*E. aerogenes* (TEM-24)	Pneumonia (64%) BSI (16%) cIAI (14%) UTI (5%) Other (0.3%)	ICU (100%)	**Clinical response**: 62% vs 70% **30-day mortality**: 33% vs 26% (ns)	No statistically significant differences in the outcome for the cefepime and carbapenem-treated groups
Chopra et al. [66]	USA (2005–2007)	Retrospective	Empiric: monotherapy *n* = 43 Definitive: monotherapy *n* = 9 (NR)	Empiric: monotherapy *n* = 14 Definitive: monotherapy *n* = 33 (NR)	*K. pneumoniae* (83%) *E. coli* (17%)	BSI (100%) -CRBSI (75%)	ICU (41%)	**In-hospital mortality** Empiric: 40% vs 36% (ns) Definitive: 33% vs 36% (ns)	Trend toward increased mortality risk with empiric cefepime therapy
Lee et al. [67]	Taiwan (2002–2007)	Retrospective	Empiric: *n* = 21 Definitive: *n* = 17 (1–2 g q8h)	Empiric: *n* = 91 Definitive: *n* = 161 (IMP 500 mg q6h, MEM 1 gr q8h, ETP 1 g q24h)	*E. cloacae* (55%) *E. coli* (24%) *K. pneumoniae* (21%)	BSI (100%) -Primary (14%) -CRBSI (21%) -Pneumonia (24%) -UTI (22%) -cIAI (16%) -SSTI (6%)	McCabe (Rapidly fatal): 11% Pitt score ≥ 4: 67%	**30-day mortality**: Definitive therapy: 59% vs 17% (p = 0.01) **Crude mortality**: 65% vs 37% (p = 0.04)	Cefepime definitive therapy inferior to carbapenem therapy, 30-day mortality was lower when cefepime MIC≤ 1 mg/L
Wang et al. [68]	USA (2006–2015)	Retrospective	*n* = 17 (1–2 g q8h)	*n* = 51 (IMP 500 mg q6h, MEM 1 gr q8h, ETP 1 g q24h)	*Klebsiella* spp. (63%) *E. coli* (32%) *P. mirabilis* (3%)	BSI (100%) -CRBSI (44%) -UTI (31%) -Biliary (9%) -Pneumonia (15%) -cIAI: (13%) -SSTI: (3%)	ICU (29%)	**14-day mortality**: 41% vs 20% (p = 0.08)	Risk of death was 2.87 times higher for patients receiving cefepime compared with carbapenems
Lee et al. [69]	Taiwan (2008–2012)	Retrospective	Definitive: *n* = 42 (1–2 g q12h or q8h)	Definitive: *n* = 53 (IMP 500 mg q6h, MEM 1 gr q8h, ETP 1g q24h)	*E. cloacae* (100%)	BSI (100%) -CRBSI (37%) -Primary (31%) -Pneumonia (9%) -UTI (8%) -cIAI: (8%) -SSTI: (6%)	McCabe (Rapidly fatal): 15% Pitt score ≥ 4: 39%	**30-day mortality**: 26.4% vs 22.2% (ns) **30-day mortality** **(bacteremia due to ESBL)**: 100% vs 42.9% (p = 0.015)	Comparison for definitive therapy due to ESBL bacteremia with cefepime SDD isolates only reported
Benanti et al. [27]	USA (2008–2015)	Retrospective	*n* = 40 (2 g q8h)	*n* = 42 (MEM 1 g q8h)	*E. coli* (100%)	BSI (100%) -CRBSI (19%) -Primary (16%) -Pneumonia (9%) -UTI (7%) -cIAI (43%) -SSTI (6%)	ICU: 26% Leukemia:79% Prior HCT: 50%	**14-day mortality**: 8% vs 19% (ns)	No difference between cefepime and carbapenem therapy
Seo et al. [23]	Korea	Randomized trial	*n* = 6 (2 g q12h)	*n* = 33 (ETP 1 g q24h)	*E. coli* (100%)	UTI (100%)	Charlson index: 5 Septic shock: 33%	**Clinical and microbiological response**: 33.3% vs 97% (*p* < 0.01)	Cefepime therapy inferior to carbapenem therapy
Suh et al. [70]	Korea (2014–2016)	Retrospective	*n* = 54 (2 g q8h or q12h)	*n* = 101 (ETP 1 g q24h)	*E. coli* (100%)	UTI (100%)	Charlson index: 2 Septic shock: 6.5%	**In-hospital mortality**: 9.3% vs 9.9% (ns)	No difference between cefepime and carbapenem therapy
Kim et al. [71]	USA (2014–2017)	Retrospective	*n* = 17 (1–2 g q12h or 2g q8h)	*n* = 89 (NR)	*E. coli* (82%) *K. pneumoniae* (18%)	UTI (100%)	ICU: 13%	**Clinical and microbiological response**: 100% vs 100% (ns) **Relapse (30-day)**: 0% vs 7%	Comparable effectiveness between cefepime and carbapenems for UTIs

BSI, blood stream infection; cIAI, complicated intra-abdominal infection; CRBSI, catheter-related blood stream infection; ETP, ertapenem; ESBL, extended spectrum β-lactamase; HCT, hematopoietic stem cell transplant; ICU, intensive care unit; IMP, imipenem–cilastatin; MEM, meropenem; NR, not reported; ns, not significant; q24h, every 24 h; q12h, every 12 h; q8h, every 8 h; q6h, every 6 h; SDD, susceptible dose dependent; SSTI, skin and soft tissue infection; UTI, urinary tract infection; vs, versus.
